# Path of intuitive compassion to transform conflicts into enduring peace and prosperity: Symmetry across domains of reiterated prisoner's dilemma, dyadic active inference, and Mahayana Buddhism

**DOI:** 10.3389/fpsyg.2023.1099800

**Published:** 2023-03-09

**Authors:** S. Shaun Ho, Yoshio Nakamura, James E. Swain

**Affiliations:** ^1^Department of Psychiatry and Behavioral Health, Stony Brook University, Stony Brook, NY, United States; ^2^Division of Pain Medicine, Department of Anesthesiology, Pain Research Center, University of Utah School of Medicine, Salt Lake City, UT, United States

**Keywords:** conflict, intuition, compassion, Mahayana Buddhism, dyadic active inference, prisoner's dilemma, *lojong*, meditation

## Abstract

Conflicts are increasingly intensified among the members of the community, making it almost impossible to extend compassion—defined as a wish to relieve others from suffering—from one side to the other, especially when both sides believe that “life is a battle of us the good *vs*. them the evil.” Is compassion even relevant to conflicts? The answer depends on how a conflict is framed in one's perception. If a conflict is perceived in a frame of zero-sum competition, then compassion is meaningless in such a “tug-of-war” mindset. Conversely, if perceived in a non-zero-sum frame—as demonstrated in reiterated prisoner's dilemma (rPD) in which two players may interdependently render win–win, lose–lose, win–lose, or lose–win scenarios by their actions—then compassion can help achieve the most preferable outcomes for all in a “dyadic dance” mindset. In this article, we present a path of intuitive compassion by pointing to symmetry across three distinct domains of rPD, dyadic active inference, and Mahayana Buddhism. In each of these domains, conflicts serve as points of bifurcation on a bidirectional path, and compassion as a conflict-proof commitment to carrying out the best strategies—even if assessed for one's own sake only—that consistently produce optimal payoffs in rPD, minimal stress in dyadic active inference, and limitless joy of ultimate enlightenment in Mahayana Buddhism. Conversely, a lack of compassion is caused by invalid beliefs that obscure the nature of reality in these domains, causing conflicts to produce even more conflicts. These invalid beliefs are produced by mistakes of over-reduction, over-separation, and over-compression in the mind, and therefore, a person's mindset is overly compressed from a multidimensional frame to a one-dimensional frame. Taken together, intuitive compassion is not about how to balance one's self-serving goals with altruistic ones. Rather, it is a conflict-proof commitment to transforming conflicts into enduring peace and prosperity according to the ultimate nature of reality. The work presented here may serve as a preliminary science-informed introduction to a genre of time-tested compassion meditations, i.e., *lojong* mind training, for the world laden with conflicts, starting from the conflicts in close relationships to those in geopolitics.

## 1. Introduction

From two kids competing for a toy to two countries competing for land, conflicts are ubiquitous in communities, either big or small. By *conflict*, we focus on any zero-sum competition between two sides in which a gain for one side means a loss for the other (so the sum for both is equal to zero). By *community*, we refer to a group of *interdependent* entities living together in a specific sphere of existence, ranging from a household to the whole planet. Of course, conflicts are only the tip of the iceberg of deeper systemic problems in a community, and therefore, conflicts will not cease until the underlying problems are addressed and cease to exist. Unfortunately, efforts to create social and economic conditions that favor cooperation and care over dominance and control are often met with great difficulty (Gilbert, 2021). There are many threats to the unity of a community, either coming from inside, such as a few elites exploiting the rest of the community in a winner-takes-it-all manner (Giridharadas, 2019), or from outside, such as disinformation campaigns by hostile foreign entities weakening the cohesion and unity at home (U. S. Department of State et al., 2022).

When more people are frustrated by various systemic problems, people will look for quick solutions to fix the problems in polarizing ways. However, actions to fix outer problems may originate from problems within our mind such that conflicts may be proliferated by those actions. Unfortunately, when community members are too occupied in in-fighting, they fail to be united against their common threats and, conversely, their common threats will exploit any in-fighting to further weaken their community. For example, people in the United States are overexposed to disinformation-saturated social media to the extent that there is no consensus on almost all public affairs, e.g., abortion right, gun right, climate change crisis, universal healthcare, vaccination, mask wearing, or even the legitimacy of the results of the 2020 Presidential election in the society. Polarization is at a historical high with deep and extensive partisan antipathy (Pew Research Center, 2014), and such divide grows even wider when facing the COVID-19 pandemic—the supposedly common threat that should have united the people (Pew Research Center, 2020). Public trust in the government has been eroding over decades, and ironically, a political party member's trust in government can go higher or lower, depending on whether the president is one of *us* or *them*, respectively (Pew Research Center, 2022). Many people in the United States seem to be influenced by the meme of “*life is a battle of us the good and them the evil*”. As this meme is a mixture of “good intention” (caring for others) and “bad idea” (at the expense of the opponent's humanity) (Lukianoff and Haidt, 2018), a firm grip of it may proliferate, rather than eliminate, conflicts.

**Figure 1:**
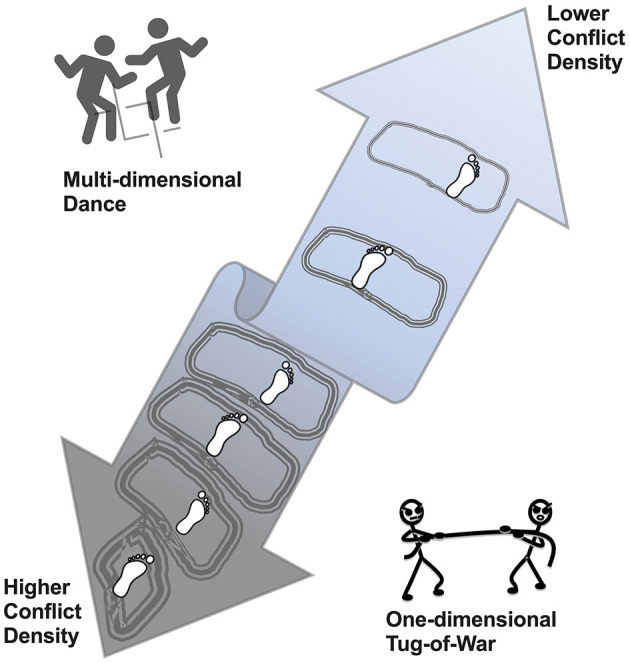
An illustration of a path for a dyadic relationship like reiterated prisoner's dilemma. The central double-arrow ribbon symbolizes the bidirectional path for movement of a dyadic relationship such as that between players in a reiterated prisoner's dilemma. Inside the ribbon, irregular shapes symbolize conflicts as stepping stones on the path, wherein each conflict means that there is a win–lose or lose–win scenario happening in the reiterated prisoner's dilemma. The footprints on those stepping stones symbolize a player's movement in a direction from bottom to top, showing that the conflict density would decrease along the way, probably due to his or her practice of Tit-for-Tat with Forgiveness strategy. The icon on the upper left corner indicates that the nature of the reiterated prisoner's dilemma is non-zero-sum, like a multidimensional dance. The icon on the lower right corner indicates that the nature of the reiterated prisoner's dilemma is misattributed to zero-sum, like a one-dimensional tug-or-war.

As problem-solving requires a community to weather through one conflict after another until underlying problems are appropriately addressed and uprooted, each conflict in the community is like a steppingstone on a bidirectional path (refer to Figure 1 for an illustration). How community members walk on each steppingstone will decide which direction they are heading on the path, either forward to a future with fewer conflicts and more peace and prosperity or backward to the opposite. To strengthen the capacity of members in a community to move in a desirable direction on the path is to give the community a fighting chance to uproot its problems underlying conflicts.

Along this line, previously, we postulated the neural basis underlying the bifurcation of conflict response in a dyadic active inference framework and introduced compassion as an intervention that aims to ensure that each conflict response is heading in the right direction (Ho et al., 2021).

**Figure 2:**
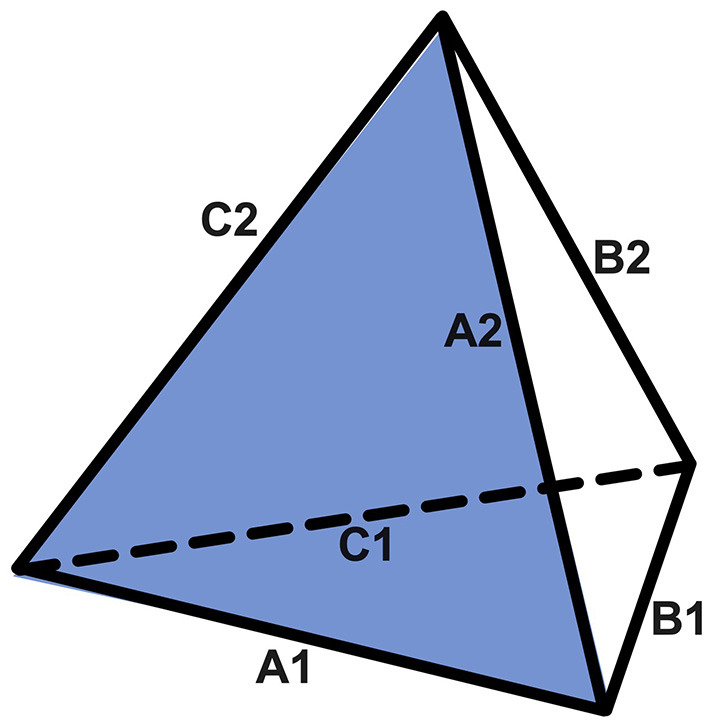
A regular tetrahedron as a geometric representation of the symmetry across domains of rPD, dyadic process, and Mahayana Buddhism. There are six sides of equal length in a regular tetrahedron, with three sides that form the bottom (A1, B1, and C1)—representing the theoretical aspects of rPD, dyadic processes, and Mahayana Buddhism, respectively—and three sides that point toward the apex of the regular tetrahedron (A2, B2, and C2)—representing the practical aspects of rPD, dyadic processes, and Mahayana Buddhism, respectively. The symmetry in the theoretical aspects is represented by the basal regular triangle that is formed by A1, B1, and C1: A1 refers to interactive payoff matrices and associated mathematical requirements in rPD; B1 refers to dyadic processes modeled as two strongly coupled active inference engines; and C1 refers to the wisdom that directly realizes the ultimate nature of reality according to Mahayana Buddhism. Likewise, the symmetry in the practical aspects is represented by those apex-oriented sides, A2, B2, and C2: A2 refers to the best winning TTF-like strategies in rPD; B2 refers to the methods to maintain a conflict-proof intersubjectivity in strongly coupled dyads; and C2 refers to a path that inseparably combines compassion and wisdom. The unity of the regular tetrahedron is made possible because all three domains are fundamentally based on the same ultimate nature of reality, i.e., effects are the interactive products of causes by conditions.

In this article, we add the Game Theory of reiterated prisoner's dilemma (rPD) (Poundstone, 1992) to our previous work and present a path of intuitive compassion (PIC) that points to a symmetry across three distinct domains, namely, rPD, dyadic active inference, and Mahayana Buddhism. We describe these domains in the following order, with a geometric form, i.e., a regular tetrahedron, to represent their theoretical and practical symmetry in Figure 2.

The first domain is the theory and practice of rPD. rPD demonstrates that mutual cooperation is not only evolutionarily plausible but also preferable under certain circumstances. In practice, a strategy, i.e., Tit-for-Tat with forgiveness (TTF), is mathematically proven to yield the most favorable outcomes in rPD. The theory and practice aspects of rPD are represented as the sides A1 and A2 of the regular tetrahedron in Figure 2, respectively.

The second domain is the theory and practice of dyadic processes. We re-introduce our dyadic active inference model and explain how invalid beliefs can hijack a person's active inference engine. In practice, we introduce key dyadic concepts underlying intersubjectivity and stress reduction that are highly analogous to TTF-like strategies in rPD. The theory and practice aspects of dyadic processes are represented as the sides B1 and B2 of the regular tetrahedron in Figure 2, respectively.

The third domain is the theory and practice of Mahayana Buddhism. We introduce classic texts by two co-founders of Mahayana Buddhism, i.e., Aryas Nagarjuna and Asanga, in the context of rPD and dyadic active inference. In practice, we introduce a genre of meditations, i.e., *lojong* mind training. Like a peacock that feeds on poisons to transform poisons into splendor, a well-versed *lojong* practitioner feeds on conflicts to transform conflicts into peace. We identify key premises underlying *lojong* practices. The theory and practice aspects of Mahayana Buddhism are represented as the sides C1 and C2 of the regular tetrahedron in Figure 2, respectively.

## 2. Theory and practice in reiterated prisoner's dilemma

The art of transforming conflicts starts from developing the discernment of zero-sum vs. non-zero-sum mindsets in which one perceives conflicts. We use a one-dimensional tug-of-war as the working metaphor for the former and a multidimensional dyadic dance for the latter. In a tug-of-war (and many sport games), the outcome of the game (winner and loser) is decided by the difference between two opposing teams' performances, so the best strategy is to *conquer* (out-perform) the opponent. In contrast, in a dyadic dance, the outcome of the game depends on the interaction between two players' games, as demonstrated in the prisoner's dilemma (Poundstone, 1992).

In prisoner's dilemma, two gang members, namely, Alice and Bob, are caught by the police, and the police do not have sufficient evidence to convict both of them on the principal charge, so they offer Alice and Bob a binary choice, either betraying their partner (Defect) or remain silent (Cooperate). The outcomes (payoffs) of Alice and Bob's plays consist of two scenarios that are fair to both Alice and Bob (a win–win and a lose–lose scenario) and two scenarios that are unfair to either Alice or Bob (a win–lose and a lose–win scenario) as follows.

1. The win–win scenario: If both Alice and Bob remain silent (Cooperate), they will receive an equal amount of payoff, e.g., both serving 1 year in prison on a lesser charge. In this case, the Payoff for Alice and Bob is denoted as (R, R), respectively, where R = −1.
2. The lose–lose scenario: If both of them betray the partner (Defect), Alice and Bob will receive an equal amount of payoff, e.g., both serving 2 years in prison, denoted as (P, P), where P = −2.
3. The win–lose scenario (unfair to Bob): If Alice defects but Bob cooperates, Alice will receive a greedy payoff (G) (e.g., be set free, G = 0) and Bob will receive an unfairly punishing payoff (U) (e.g., serve 3 years in prison, U = −3). The payoff for Alice and Bob is denoted as (G, U), respectively, where (G, U) = (0, −3).
4. The lose–win scenario (unfair to Alice): If Alice cooperates but Bob defects, Alice will serve 3 years in prison (U) and Bob will be set free (G), denoted as (U, G), where (U, G) = (−3, 0).

The generalized payoff matrix is listed in Table 1. The values in the payoff matrix follow the order:


$$\begin{array}{l} \text{G}>\text{R}>\text{P}>\text{U} \end{array}$$

Note that in the example given above, the payoff is measured as (−1) times the number of years to serve in prison, i.e., (G, R, P, U) = (0, −1, −2, −3).

**Table 1:** Generalized payoff matrix of prisoner's dilemma.

**Payoff matrix [Payoff**_**(Alice)**_ **, Payoff**_**(Bob)**_ **]**		**Bob's play**	
		**Cooperate**	**Defect**
Alice's play	Cooperate	Win-win (R, R)	Lose-win (U, G)
	Defect	Win-lose (G, U)	Lose-lose (P, P)

When the same two players play PD repeatedly over time and they can remember the opponent's immediately preceding play, as in a Markov chain, rPD is at play. An additional requirement


$$\begin{array}{l} \text{2R}>\text{G}+\text{U} \end{array}$$

is needed to make rPD in favor of the win–win scenario relative to other scenarios. This additional requirement “makes the pie bigger” for the win–win scenario than that in the win–lose and lose–win scenarios, as the sum of the payoffs for Alice and Bob in the win–win scenario (2R) is greater than those in the win–lose and lose–win scenarios (G + U). For each player, this additional requirement can make the repetition of mutual cooperation (i.e., expected payoff = R) more preferable to alternating between win–lose and lose–win indefinitely [i.e., expected payoff = (G + U)/2]. *This additional requirement is the key that leads to enduring peace—meaning that there is no cyclic conflict between Alice and Bob—and prosperity— meaning that both Alice and Bob will gain more than otherwise—in rPD*.

In the prisoner's dilemma, the payoff is not solely determined by one's own play unilaterally, as Axelrod stated:

“*...what is best depends in part on what the other player is likely to be doing. Further, what the other is likely to be doing may well depend on what the player expects you to do*.” (Axelrod, 1984)

Essentially, a player's payoff in rPD is consistent with the notion in Madhyamaka Buddhist Philosophy that effect is an interactive product of cause by condition (Ho et al., 2022), denoted as follows.


$$\begin{array}{l} \text{Effect}=\text{Cause}\times \text{Condition} \end{array}$$

Here, the effect is one player's payoff, the cause is the player's own play (Cooperate or Defect), and the condition is the opponent's play (Cooperate or Defect). So, the payoff for Alice and Bob is denoted as


$$\begin{array}{l} {\text{Payoff}}_{\left(\text{Alice}\right)}={\text{Play}}_{\left(\text{Alice}\right)}\times {\text{Play}}_{\left(\text{Bob}\right)}\\ {\text{Payoff}}_{\left(\text{Bob}\right)}={\text{Play}}_{\left(\text{Bob}\right)}\times {\text{Play}}_{\left(\text{Alice}\right)} \end{array}$$

In practice, there are several archetypical strategies in playing rPD, including Random, Cooperator, Defector, Alternator, Nice-unless-Grumpy, and Tit-for-Tat. In Random, the play of Cooperate or Defect is chosen randomly. In Cooperator, the player always cooperates. In Defector, the player always defects. In Alternator, the player alternates between cooperating and defecting. In Nice-unless-Grumpy, the player defects only after a certain level of grumpiness that increases when the opponent defects and decreases when the opponent cooperates. In Tit-for-Tat, the player starts with a cooperative play and, starting the second trial, its current play (“tit”) simply mimics what the opponent did the last time (“tat”). When different strategies are pitted against each other in tournaments repeatedly, Tit-for-Tat robustly emerges as the winning strategy over and over again, demonstrating the value of (1) not being the first to defect, (2) being somewhat forgiving, and (3) being provokable in the sense that the opponent's first defection will be surely retaliated by choosing the play of Defect (Axelrod, 1980a,b).

However, even if both players jointly adopt the Tit-for-Tat strategy, they are prone to a “death spiral” where a one-time, single-bit error in either player's play, e.g., when one agent defects and the opponent cooperates, will lead to a never-ending alternating scenario between cooperation and defection, yielding a lower expected payoff, (G + U)/2, than the expected payoff, R, of repeated mutual cooperation. To escape this “death spiral”, a strategy called “Tit-for-Tat with Forgiveness” (TTF) can be employed. In this modified strategy, when the opponent defects, a player employing this TTF strategy will occasionally cooperate on the next play despite the opponent's play on the previous trial, and the exact probability that a player will forgive the opponent's defection depends on his or her opponent's behaviors. To maintain the reciprocity, the opponent's very first defection will not be forgiven in TTF.

TTF-like strategies in rPD appear to have the following features (Axelrod, 1984):

1. *Nice*: A successful player shall not be the first to defect. This feature prevents the player from getting into unnecessary trouble.
2. *Reciprocating*: A successful player must reciprocate both cooperation and defection, and therefore, it should be provoked by the very first defection by the opponent and consequently retaliate against the opponent's defection in the previous play, except occasional forgiveness. This feature discourages the opponent from persistently trying to defect.
3. *Forgiving*: A successful player must also be forgiving sometimes, despite the fact that the opponent just defected in the previous play. This feature helps restore mutual cooperation and reduce the likelihood that both parties will get into long runs of revenge and counter-revenge.
4. *Non-envious*: A successful player is not envious of the other player's success, i.e., not striving to score more payoff than the opponent.

Due to the clarity of the behaviors of a player employing TTF, other players in a tournament will come to adapt to TTF as well (Axelrod, 1984). The *contagiousness* of TTF has been corroborated in rPD tournaments that used machine-learning algorithms to simulate the evolution of rPD (Surma, 2019).

In summary, when conditions are suitable (i.e., G > R > P > U and 2R > G + U in the payoff matrix), TTF-like strategies are not only evolutionarily plausible but also robustly preferable for both players to earn as much payoff as possible in practicality, as noted in the book “the evolution of cooperation” (Axelrod, 1984):

“*If a nice strategy, such as TIT FOR TAT, does eventually come to be adopted by virtually everyone, then individuals using this nice strategy can afford to be generous in dealing with any[sic] others. In fact, a population of nice rules can also protect itself from clusters of individuals using any other strategy just as well as they can protect themselves against single individuals... These results give a chronological picture for[sic] the evolution of cooperation. Cooperation can begin with small clusters. It can thrive with rules that are nice, provocable, and somewhat forgiving. And once established in a population, individuals using such discriminating strategies can protect themselves from invasion. The overall level of cooperation tends to go up and not down. In other words, the machinery for the evolution of cooperation contains a ratchet*.” (Axelrod, 1984)

Based on the analysis described earlier, we postulate the following hypothesis:

**Hypothesis 1**: *When two players are engaged in an rPD-like relationship, a player's commitment to following TTF-like strategies will ensure the possibility to transform conflicts (alternating between win–lose and lose–win scenarios) into enduring peace and prosperity (repeated win–win scenarios)*.

## 3. Theory and practice in dyadic active inference

Symmetrically, the four properties of TTF-like strategies, namely, nice, reciprocate, forgiving, and non-envious, are highly consistent with the principles that we have identified in our active inference framework for dyadic interactions, i.e., maintaining symbiotic benevolence and mitigating problems of under-coupling and over-mentalizing to promote stress reduction, compassion, and intersubjectivity, and we have elucidated underlying neural and theoretical bases in a series of articles (Ho et al., 2020, 2021, 2022). For a very brief introduction of dyadic active inference, refer to Box 1. We hereby summarize the take-home messages from our previous work and then refute the validity of the meme of “us the good vs. them the evil” accordingly.

Box 1 **Dyadic active inference framework.**

As described with more details previously (Ho et al., 2022), according to Free-Energy Principle (FEP), a living organism is a self-organizing system that maintains its characteristic phenotypic states and avoids surprising deviations from these expected states by generative processes that are self-organizing and self-evidencing (Friston, 2013; Ramstead et al., 2020; Friston et al., 2022). As the physical, biological processes of an organism embody its “best guess” about its environments, on average and over time, the organism tends be attracted to a limited number of attractor states in the space of all possible states, with low entropy or spread in the probability density over the space of possible states, i.e., low variational free energy. Variational free energy is a measure of the upper bound of surprise or prediction error—the difference between the organism's “best guess” beliefs about what caused its sensory states and what it observes. The FEP leverages the principle of surprise minimization to optimize the prior beliefs in the active inference engine by minimizing variational free energy—the upper bound of surprise. There are two ways to minimize variational free energy, namely, perceptual inference and active inference. In perceptual inference, agents strive to update their prior beliefs, while in active inference, agents change their environment (or their sampling of information from the environment) by selecting a plan or policy in a set of prior beliefs that would yield the least expected free energy (Peters et al., 2017). Notably, in FEP, the variational free energy is more of a function of beliefs and expectations in the internal states, rather than a function of the environments hidden from the internal states (Ramstead et al., 2020). In such processes, internal and active states' dynamics are a function of, and only of, a variational free energy bound on surprise, and the belief optimization is implicitly done in the minimization of variational and expected free energy (Friston et al., 2022).

The notion of active inference emphasizes that actions solicit a sensory outcome that informs approximate posterior beliefs about external states of the world. Such generative process in FEP renders a living organism to be participatory, or enactive in soliciting and, therefore, co-creating its perception of the external states, which is very different from a representationalist process by which external states generate sensory states exclusively (Friston et al., 2022). Heuristically, one may consider that an active inference engine is actively self-evidencing what the world should be (known as an enactive account), rather than passively learning to represent what the world seems to be (known as a representationalist account)—a distinction that has been elaborated in the literature (Ramstead et al., 2020).

**Figure 3:**
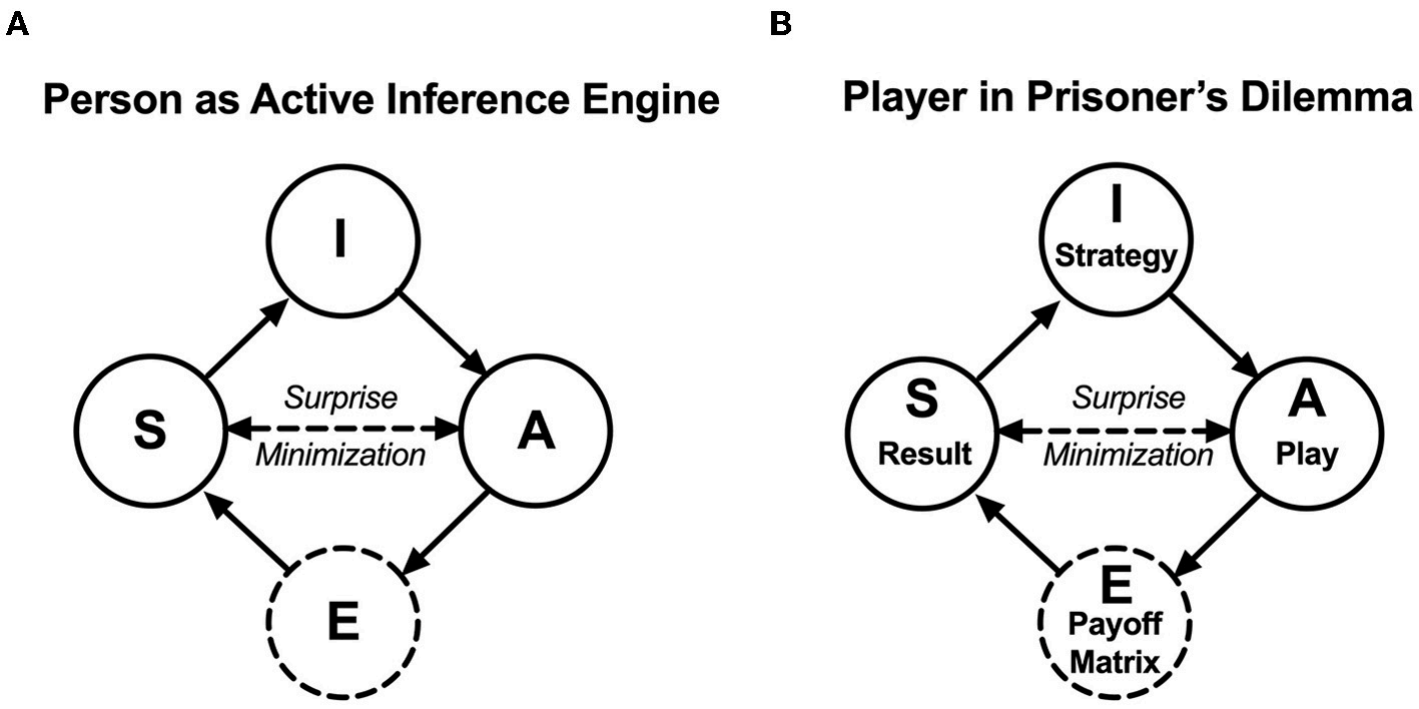
**(A)** An active inference model and its environments: in an active inference model, an adaptive person functions as an active inference engine—consisting of nodes Active State (A), Sensory State (S), and Internal State (I) (solid circles)—interacting with external events in the environments, node External State (E) (dashed circle). In a hierarchical network, (E) represents events from environments at an external level, (S) represents the person's afferent sensory state, and (A) represents the person's efferent active state, both at a lower level, and (I) represents the person's prior beliefs at a higher level. Nodes (E) and (I) do not have direct effects on one another, as they are separated by nodes (A) and (S). The double-arrowed line between (A) and (S) indicates the notion of active inference, that actions solicit a sensory outcome that informs approximate posterior beliefs in the internal states (I) about the external states (E). This is done by minimizing variational free energy—the upper bound of surprise of the active inference. **(B)** Applying the active inference model to reiterated prisoner's dilemma, the nodes (E), (S), (A), and (I) can be equivalent to (Payoff Matrix of rPD), (Result, i.e., the readout of payoff in a trial), (Play of “cooperate” or “defect”), and (Strategy, e.g., Tit-for-Tat), respectively.

Inspired by FEP (Friston, 2013), we suggest that a person can be formally modeled as an active inference engine in a multi-level network consisting of four nodes, namely, nodes of sensory states (S), active states (A), internal states (I), and external states or events (E). This network is partitioned into an external state (E) and an active inference engine that consists of the nodes (S) and (A) at a lower level and node (I) at a higher level (see Figure 3.

**Figure 4:**
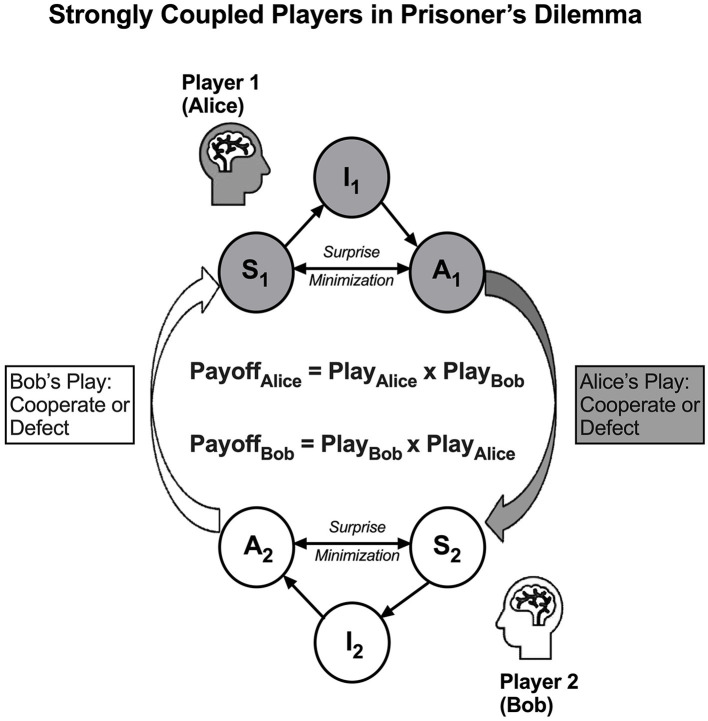
Dyadic active inference model in reiterated prisoner's dilemma: when two players (Alice as Player 1 and Bob as Player 2) are strongly coupled, one person's active states outputs become the total inputs of the other person's sensory states, and vice versa, i.e., (A_1_) causes (S_2_) and (A_2_) causes (S_1_), the surprise in Persons 1 and 2 are also coupled and thus the strategies in their internal states (I_1_ and I_2_) are optimized collectively. The results (S_1_ and S_2_), i.e., payoffs, are determined by the interaction between the players' plays (A_1_ and A_2_), i.e., Payoff_Alice_ = Play_Alice_ × Play_Bob_ and Payoff_Bob_ = Play_Bob_ × Play_Alice_, as if they are performing a multidimensional dyadic dance.

We need a dyadic active inference model of two agents that are strongly coupled to model dyadic interactions. Just like ice and water are two phases of the same H_2_O molecules that behave distinctly (solid and liquid, respectively), the same active inference engine can behave very differently between the phases of weakly coupled and strongly coupled states—while an active inference engine maintains conditional independence between its internal and external states in a weakly coupled state, such conditional independence is diminished in a strongly coupled state, when its external states are no longer a unitary node (E), but rather another active inference engine, such that one engine's active states (A) serve as a primary source of input to the other engine's sensory states (S), and vice versa. In the most strongly coupled state, one person's active states will become total environmental inputs for the other person's sensory states, and vice versa (see Figure 4).

**Figure 5:**
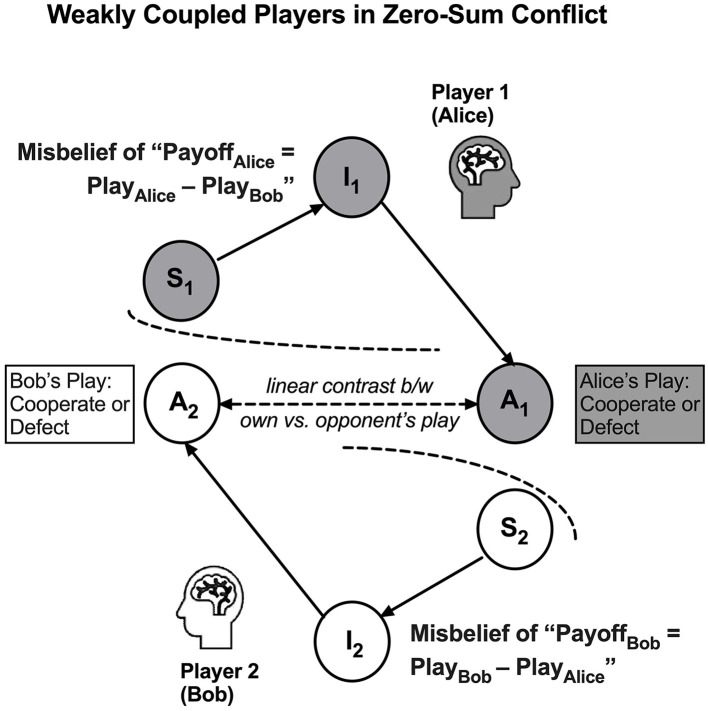
An under-coupling ensues in a dyadic system when the players become blind to one another's active inference engine, especially those covert, sensory, and internal states (S_2_) and (I_2_). Due to the under-coupling, the players tend to misattribute that the results are determined by the linear contrast between their plays, i.e., misbeliefs of Payoff_Alice_ = Play_Alice_ – Play_Bob_ and Payoff_Bob_ = Play_Bob_ – Play_Alice_, as if they are competing in a zero-sum one-dimensional tug-of-war.

According to our previous work (Ho et al., 2020, 2022), we have identified three inter-related problems that that may impair dyadic interactions that, fortunately, can be mitigated by effective dyadic interventions: (1) *deficient relational benevolence due to invalid beliefs, (2) under-coupling, and (3) over-mentalizing*, as follows:

1) *Deficient relational benevolence:* invalid beliefs prevents the awareness of relational benevolence. As depicted in Figure 4, when two persons (e.g., Alice and Bob as Players 1 and 2 in rPD) are strongly coupled (A*_1_* ≈ S*_2_* and A*_2_* ≈ S*_1_*), the variational free energy is minimized collectively *if*, and *only if*, the surprise (prediction errors) in one person is minimized without increasing the other's. Therefore, Player 1 can achieve intersubjectivity by minimizing his or her variational free energy through communicative interactions with Player 2, wherein Player 1's prior belief would approximate Player 2's prior beliefs (I_1_ ≈ I*_2_*). We have postulated that invalid beliefs (*Vikalpas*) will diminish the awareness of relational benevolence and of the prior beliefs of each person's active inference engine (Ho et al., 2021).

2) *Problem of under-coupling:* Under-coupling increases variational free energy. As depicted in Figure 5, when Player 1 engages Player 2's overt behaviors only, Player 1 may reduce Player 2, who serves as Player 1's external states, to a unitary object without its own inner states such as feelings and prior beliefs. Thus, Player 1 would fail to achieve intersubjectivity and find it difficult to reduce stress in either party. For example, when Alice neglects to see that her plays cause Bob to feel negatively and only focuses on how to out-perform Bob, Alice would fail to recognize Bob's attempts to reduce Bob's own variational free energy and therefore Alice's variational free energy during dyadic interactions would increase.

3) *Problem of over-mentalizing:* Over-mentalizing can perpetuate impairments of dyadic interactions. Over-mentalizing can happen when cyclic conflicts render players defensive against one another repeatedly and, therefore, misattributing the other player's defections to malice or character flaw rather than his or her ignorance of the best strategies that involve reciprocal benevolence. Conceptual thoughts (*Vikalpas*) are responsible for the problem of over-mentalizing.

### 3.1. Summary of take-home messages from our previous work

#### 3.1.1. A person is an active inference engine

An active inference model is a formal model postulating that a living entity, e.g., a person, is functionally an active inference engine that strives to adapt to the environment by minimizing variational free energy that arises through surprises during person–environment interactions. In its simplest form, the person–environment interactions can be modeled as interactions between external states and the person, and the person can be modeled as an active inference engine consisting of sensory states, active states, and internal states. The sensory and active states of an active inference engine serve as an interface with the environments, including another person in dyadic interaction. The internal state does not directly interact with the environment and contains prior beliefs, plans, policies, or strategies that are updated and optimized through a surprise minimization process (Ho et al., 2022). Refer to Figure 3A for an active inference model of a person and Figure 3B for a heuristic application of this model to a player in PD.

#### 3.1.2. Dyadic coupling between active inference engines and emerging conditional independence between self and other

Parent–child interactions are essential for the development of a person, which means that dyadic processes (person–person interactions) are key to the development of an active inference engine. Although the duality of self and others emerges as a result of apparent conditional independence between two active inference engines, all persons and their environments are functionally connected interdependently when they are placed in a strongly coupled state. Thus, the apparent duality—a person who exists independently of the rest of the world—is just an illusion (Ho et al., 2022). As it is explained later, not realizing the illusory conditional independence between self and others in dyadic interactions is a mistake of *over-separation*.

#### 3.1.3. Two states of an active inference engine

Active inference engines can appear to function in two distinct states, namely, a strongly coupled state and a weakly coupled state (Ho et al., 2022). When two persons' active inference engines are entangled in the strongly coupled state, the input to one person's sensory states is predominantly coming from the output from the other person's active states, and vice versa. When the surprise is minimized during this strongly coupled state, one person's internal states are approximating the other person's internal states, reaching a high level of intersubjectivity—subject–subject understanding of covert events of one's intentions or feelings (Ho et al., 2022). A high level of intersubjectivity enables two persons to understand one another's internal beliefs, plans, policies, or strategies underlying their overt behaviors. Notably, due to the strong coupling, the dyad will imitate one another's actions in a way that is the hallmark of TTF-like strategies in rPD. Refer to Figure 4 for a heuristic model of two active inference engines that are strongly coupled in the context of rPD. Conversely, when an active inference engine is not strongly coupled with another active inference engine, it will reside in a weakly coupled state. In the weakly coupled state, an active inference engine will perceive the world from a self-centered, egoistic perspective, as if the observer were independent of the observed objects that are not relationally interacting with the observer (Ho et al., 2022).

#### 3.1.4. The active inference engine is hijacked by invalid beliefs in dyadic processes

While it is normal for an active inference engine to alternate between a strongly coupled state and a weakly coupled state, it would be a problem if a person fails to establish sufficient intersubjectivity during its strong coupling with another person. If an active inference engine fails to maintain the strong coupling in a dyadic interaction, the surprise, which is proportional to the stress perceived by an active inference engine (Peters et al., 2017), will become excessive and, therefore, harmful to the dyad (Ho et al., 2022). We have proposed that invalid beliefs play a key role when they hijack the active inference engine, causing problems of under-coupling and over-mentalizing during the time of dyadic interactions (Ho et al., 2022). We postulated that invalid beliefs are nothing but invalid conceptual thoughts (*vikalpas* in Sanskrit) that are enshrouded in our systems by the working of mental fabrication/proliferation (*prapañca* in Sanskrit) (Ho et al., 2021), described later.

#### 3.1.5. A hijacked active inference engine is burdened with excessive stress and weariness

It has been established that chronic stress can accelerate mental and physical weariness and aging (McEwen, 2012). Furthermore, stress can be conceived as a result of excessive variational free energy in an active inference engine (Peters et al., 2017). Stress arising in dyadic interactions can become excessive if an active inference engine is hijacked by invalid beliefs (Ho et al., 2020). Although stress in dyadic interactions can interfere with decision-making (Ho et al., 2014) and caring behaviors (Kim et al., 2015), it can also be mitigated through dyadic interventions, as supported by our neuroimaging studies (Swain et al., 2017; Ho et al., 2020) and systematic review on the efficacy of parenting interventions on parenting stress (Ho et al., 2022).

#### 3.1.6. The post-conflict bifurcation between two incompatible paths

Previously, we identified an entry point of bifurcation between two incompatible post-conflict responses for an individual: (a) attuning to the counterparts' perspectives and needs *despite* the conflicts and (b) blocking the attunement to the counterparts *due to* the conflicts. The post-conflict paths diverge depending on the presence or absence of the valid view of the ultimate nature of reality—that the conflict and its solution are effects of an interactive product of cause by condition. In our previous work, we described the potential neural basis underlying these two paths (Ho et al., 2021). We describe the post-conflict bifurcation in the context of rPD later.

### 3.2. Refuting “*life is a battle of us the good vs. them the evil*”

Here we conduct a logical analysis to refute the notion of “*life is a battle of us the good vs. them the evil*”. According to the work by Arya Asanga (circa 380 CE), there are eight types of conceptual thoughts (*vikalpas* in Sanskrit) and three kinds of mental fabrication/proliferation processes (*prapañcas* in Sanskrit) that obscure the realization of ultimate reality (Asanga, 2016, p. 89–96), as follows.

The eight types of invalid conceptual thoughts (*vikalpas*) are as follows:

Type 1. The conceptual thought that conceives of an essential nature.
Type 2. The conceptual thought that conceives of a distinguishing characteristic.
Type 3. The conceptual thought that grasps a collection (of distinguishing characteristics) as a separate entity.
Type 4. The conceptual thought that conceives of an “I”.
Type 5. The conceptual thought that conceives of entities as being “mine”.
Type 6. The conceptual thought that conceives of entities as being agreeable.
Type 7. The conceptual thought that conceives of entities as being disagreeable.
Type 8. The conceptual thought that conceives of entities as being neither agreeable nor disagreeable, thus leading to an attitude of indifference toward it.

The three levels of mental fabrication/proliferation processes (*prapañca*) are as follows:

Level I. This level of substance provides a basis for the first three types of *vikalpas* (Types 1–3). These three types are beliefs that property exists deterministically in a specific form, identical to its observed appearance, independent of specific circumstances. This substance serves as the basis of *prapañca*, a proliferating process that superimposes ego onto an impersonal process, through which the next levels of substances, and the *vikalpas* supported by them, develop.
Level II. This level of substance is the basis of the next two types of *vikalpas* (Types 4–5). The view of ego is one that erringly grasps a self that is separate from a collection of perishable events, i.e., the “I” who affirms itself to exist, which is the root of all egoistic views. The egoistic views feed an egoistic conceit—a sense of entitlement to justify an event's value as “good” or “bad” according to one's own views, self-affirmingly, e.g., “It's good or bad (because I think so)”.
Level III. This level of substance is the basis of the last three types of *vikalpas* (Types 6–8)—which evaluate entities as being agreeable, disagreeable, or neither and give rise to craving, hatred, or ignorance according to circumstances, respectively.

We postulate that the notion of “*us the good vs. them the evil*” may be caused by three major mistakes corresponding to the three levels of *prapañcas*, namely, *over-reduction, over-separation, and over-compression*, which obscure the direct realization of the ultimate nature of reality, described below.

#### 3.2.1. Over-reduction

In accordance with the first level of *prapañca* and the first three types (types 1–3) of *vikalpas*, over-reduction is the most subtle mistake among the three major mistakes discussed here. Over-reduction results from the failure to recognize as many variables as there are operating in dyadic interactions, which makes it impossible for a person to perceive the dependent origination nature of the dyadic interactions.

#### 3.2.2. Over-separation

In accordance with the second level of *prapañca* and the middle two types (types 4–5) of *vikalpas*, over-separation dichotomizes all phenomena into categories of “I/mine” vs. “not I/not mine”. The two categories are overly disjointed because the inter-dependent relationship between them is ignored. An over-separation will mislead a player in rPD to think that his or her own payoff will not be an effect of the interactive product of one's own play by the opponent's play.

#### 3.2.3. Over-compression

In accordance with the last level of *prapañca* and the last three types (types 6–8) of *vikalpas*, over-compression confounds multiple dimensions of ownership, actions and feelings, and the outcomes (payoff) in rPD. Over-compression will mislead a player to erroneously think that his or her payoff is a function of a linear contrast between two competing players, pursuing the maximal difference. Obviously, this kind of zero-sum thinking is contrary to the “non-envious” quality in TTF-like strategies. As the differential payoff of own vs. opponent's is positive in the win–lose scenario and zero in the win–win scenario, the player would overestimate the expected payoff of the unfair win–lose scenarios (I win vs. opponent loses), thinking “*what if I can just exploit the opponent and then run away from it*”, and, at the same time, the player would underestimate the expected payoff of the win–win scenario if he or she would choose to employ TTF-like strategies.

This logical refutation can be expressed formally based on the fact that, as described above, the payoffs in rPD are the effects of the *non-additive* interaction between two players' actions. If Alice and Bob are trapped by the misbelief of “*us the good vs. them the evil*”, they will misperceive their payoffs to be an additive function of their plays, denoted below:


$$\begin{array}{l} {\text{Payoff}}_{\left(\text{Alice}\right)}={\text{Play}}_{\left(\text{Alice}\right)}-{\text{Play}}_{\left(\text{Bob}\right)}\\ {\text{Payoff}}_{\left(\text{Bob}\right)}={\text{Play}}_{\left(\text{Bob}\right)}-{\text{Play}}_{\left(\text{Alice}\right)} \end{array}$$

If laden with such a misperception, Alice and Bob would compete to conquer one another in a zero-sum frame, that is,


$$\begin{array}{l} {\text{Payoff}}_{\left(\text{Alice}\right)}+{\text{Payoff}}_{\left(\text{Bob}\right)}=0 \end{array}$$

Such a “tug-of-war” mindset leads them to either cyclic conflicts or endless lose–lose scenarios in rPD. Refer to Figure 5 for a heuristic model of two active inference engines in the “tug-of-war” mindset. Being trapped in cyclic conflicts, worn-out players would not value the utility of being nice, reciprocating, forgiving, and non-envious of others' success and, therefore, forsaking any possibility of employing TTF-like strategies.

Taken together, we postulate the second hypothesis of the present study:

**Hypothesis 2**: *As a result of the mistakes of over-reduction, over-separation, and over-compression, a player in rPD may mistakenly believe that payoff is a linear function of the players' plays*.

### 3.3. Practical dyadic concepts in dyadic processes

Previously, we have applied our dyadic active inference framework to make sense of the efficacy of parenting interventions for reducing parenting stress (Ho et al., 2022), wherein we suggested that the strong coupling state between two active inference engines can parsimoniously explain nine dyadic concepts that have emerged in the practice of dyadic processes (Provenzi et al., 2018), as follows:

Mutuality: Mutual contribution of the interactive partners.
Reciprocity: Reciprocal influence between interactive partners.
Attunement: Recognition of one another's intentions underlying actions.
Contingency: Timely, reciprocal adjustment of affective and behavioral signals.
Coordination: Bidirectional rhythmic exchanges characterized by specific timing and turn-taking, which facilitates the reciprocal prediction of future behavioral states.
Matching: Simultaneous exhibition of the same affective and/or behavioral state.
Mirroring: Exaggerated/marked imitation of trans-modal affective quality in a temporally contingent way.

Reparation: Transforming unmatched dyadic states to matched dyadic states producing an opportunity to learn interactive strategies and to achieve better stress and emotion regulation.
Synchrony: Degree of congruence between trans-modal behaviors of two partners that lagged in time.

All of these dyadic concepts are applicable to rPD. First and foremost, because the requirements of rPD's payoff matrix favor mutual cooperation, rPD is consistent with Mutuality and Reciprocity outright. Furthermore, the recognition of the opponent's strategy in rPD reflects Attunement (to one another's intentions). When both players in rPD adapt to employ TTF-like strategies, their “Tit for Tat” can be described as the dyadic concepts of Mirroring (imitation of the partner's last play), Contingency (between the opponent's last play and one's own current play), and Synchrony (as the coherence between plays on both sides over time). When their plays are identical in the win–win or lose–lose scenarios, it is consistent with Matching (the same play simultaneously). The retaliation of a player against the opponent's surprising defection and the return to cooperation if the opponent renews his or her cooperation are consistent with Reparation (by discouraging defections in the future), Reciprocity, and Contingency. The occasional forgiveness of the opponent's defections facilitates the reinstatement of mutual cooperation, which is key to Reparation.

### 3.4. Two examples of rPD in close relationships

Here we discuss two examples of rPD analysis in partner–partner relationships. We discuss the application of Mahayana Buddhist meditation to partner–partner relationships later.

For example, Alice and Bob are partners. They will be happy if their daily interactions yield win–win scenarios most of the time, with the sporadic win–lose or lose–win scenarios that may happen from time to time. In general, a happy partnership will be sustained by both partners if they will exhibit behaviors that are nice, reciprocating, forgiving, and non-envious (or non-dominating), all of which are consistent with TTF-like strategies. Conversely, they will be unhappy if they often find themselves trapped in lose–lose scenarios or constantly alternating win–lose and lose–win, but rarely experience win–win scenarios together due to the lack of forgiveness and reciprocity that are crucial to the reparation of an ongoing relationship.

We assume that Alice and Bob's relationship can be simplified to have a payoff matrix that meets the two requirements, G > R > P > U and 2R > G + U, to qualify their intimate relationship as rPD. Each of them has two binary plays, Conjoin and Dissociate, which are mutually incompatible, with the former defined as any actions that will strengthen their togetherness or shorten their distance and the latter defined as any actions that will weaken their togetherness or lengthen their distance.

There are two versions of rPD for Alice and Bob as partners, depending on how the payoff matrix is defined in their relationship. In the first version, Conjoin is functionally equivalent to Cooperate, and Dissociate is functionally equivalent to Defect, such that the payoff of win–win scenario [Payoff_(Alice)_, Payoff_(Bob)_] = (R, R) is attained if both players Conjoin, and the payoff of lose–lose scenario [Payoff_(Alice)_, Payoff_(Bob)_] = (P, P) is attained if both players Dissociate, as denoted in Table 2. If both players follow TTF-like strategies, then their union will be favored.

**Table 2:** The payoff matrix of Alice and Bob's relationship in favor of union.

**Payoff matrix [Payoff**_**(Alice)**_ **, Payoff**_**(Bob)**_ **]**		**Bob's play**	
		**Conjoin**	**Dissociate**
Alice's play	Conjoin	Win-win (R, R)	Lose-win (U, G)
	Dissociate	Win-lose (G, U)	Lose-lose (P, P)

In the second version, Conjoin is functionally equivalent to Defect, and Dissociate is functionally equivalent to Conjoin, such that the payoff of win–win scenario [Payoff_(Alice)_, Payoff_(Bob)_] = (R, R) is attained if both players Dissociate, and the payoff of lose–lose scenario [Payoff_(Alice)_, Payoff_(Bob)_] = (P, P) is attained if both players Conjoin, as denoted in Table 3. If both players follow TTF-like strategies, then their break-up will be favored.

**Table 3:** Payoff matrix of Alice and Bob's relationship in favor of divorce.

**Payoff matrix [Payoff**_**(Alice)**_ **, Payoff**_**(Bob)**_ **]**		**Bob's play**	
		**Dissociate**	**Conjoin**
Alice's play	Dissociate	Win-win (R, R)	Lose-win (U, G)
	Conjoin	Win-lose (G, U)	Lose-lose (P, P)

Whether the win–win scenario means that both players do the same action of one kind (Conjoin) or the other (Dissociate) depends on whether both players agree to consider union or divorce to be their win–win scenario. Thus, although these two examples do not have the same outcome in terms of union or divorce, they are the same in terms of yielding robustly favorable win–win scenario's payoff (R, R) for the dyad.

## 4. Theory and practice of Mahayana Buddhism

Mahayana Buddhism (a.k.a. Great Vehicle Path to Enlightenment) represents a path to transform conflicts into enduring peace and prosperity, with a commitment to the fulfillment of self and others' aims *equally*. The combination of compassion and wisdom is a common quality throughout the base, path, and ultimate attainment of Mahayana Buddhism, as stated by one of the greatest Tibetan Buddhist teachers, Gelek Rimpoche (1939–2017):

“*The essence of compassion is wisdom*.

*The essence of wisdom is compassion.”*

Accordingly, we hereby coin the term *intuitive compassion* to refer to wisdom that is not separate from compassion and compassion that is not separate from wisdom. Mahayana Buddhism cultivates intuitive compassion gradually in the following order:

1. Compassion (and benevolence),
2. Great compassion,
3. Conventional enlightenment-oriented mind, and
4. Ultimate enlightenment mind.

According to Buddhist definitions, compassion (*karuṇā* in Sanskrit) refers to the wish that others be free from suffering, and benevolence (*maitri* in Sanskrit) refers to the wish that others be happy (Buswell and Lopez, 2013). Compassion is a seed for great compassion (*mahākaruṇā* in Sanskrit), which is defined as the wish to free *all sentient beings* from suffering, which is distinguished from compassion by its scope (all sentient beings) and its agency (one personally seeks to alleviate the suffering of all other beings) (Buswell and Lopez, 2013). Great compassion is a seed for a conventional enlightenment-oriented aspiration (*bodhicitta* in Sanskrit) that propels those so-called enlightenment-oriented sentient beings (*bodhisattvas* in Sanskrit) to attain the wisdom that can enable them to fulfill self and others' aims *equally* (Buswell and Lopez, 2013). The combination of sufficient wisdom and *conventional bodhicitta* will enable the practitioner to attain the *ultimate bodhicitta* (*paramārthabodhicitta* in Sanskrit), which refers to the bodhisattva's direct realization of the ultimate truth (Buswell and Lopez, 2013). Ultimately, the practitioner who completes this path will attain the inseparable union of compassion and wisdom—intuitive compassion. We consider intuitive compassion a functional synonym to ultimate bodhicitta, for the reasons provided later.

One way to understand this developmental process is to note that the ever-broadening scope of compassion in this graded path parallels the ever-broadening scope of one's *identification*—*treating someone's conditions as if one's own without discrimination*. In the beginning, a practitioner can only identify with those near and dear to him or her, such as a parent's natural compassion and love for his or her child, which is supported by neurobiological factors (Swain and Ho, 2017; Swain et al., 2019; Eslinger et al., 2021). Then, progressively, he or she can identify with those “*friends”* who support his or her interest, then with those “strangers” who seem unrelated to his or her interest, then with those “enemies” who are in conflict with his or her interest. Thus, eventually, the practitioner identifies with all sentient beings *equally*. With each and every person that he or she identifies with additionally, the practitioner maintains a commitment to attaining self and other's aims through his or her own work of compassion (when the aim is to be free from suffering) and benevolence (when the aim is to be happy). As such commitment continuously calls for more know-how to solve more problems that are brought on by *identifying* with an ever-increasing number of people, the practitioner aspires the total enlightenment to ensure that he or she can complete the path. In this way, the ever-expanding identification with others will propel the practitioner to accumulate more merits conducive to intuitive compassion along the way.

From this perspective, it becomes clear that the practitioners who are committed to developing intuitive compassion will accept a premise that is not accepted otherwise, which is stated in the following hypothesis:

**Hypothesis 3**: *A PIC practitioner will identify with every sentient being that he or she engages, seeing them as someone with whom he or she has been playing rPD indefinitely, together with an invariant commitment to transform conflicts into enduring peace and prosperity, regardless of their current relationship as friends, strangers, or enemies*.

This hypothesis can find support in a pithy summary of Mahayana Buddhism composed by Jetsün Drakpa Gyaltsen (1147–1216), entitled Parting from Four Attachments, as follows:

“If you are attached to this life, you are not a true spiritual practitioner
If you are attached to *saṃsāra*, you have no renunciation
If you are attached to your own self-interest, you have no *bodhicitta*
If there is grasping, you do not have the (proper) view.”

The attachment here refers to the eighth or ninth of the twelve links of dependent origination (*pratityasamutpāda* in Sanskrit), namely, craving (*tṛṣṇā* in Sanskrit) or clinging/attachment (*upādāna* in Sanskrit), respectively, and it is followed by the tenth link, becoming (*bhava* in Sanskrit). Craving is defined as the desire to keep a feeling of pleasure or to separate from a feeling of pain, or as a non-diminution of a neutral feeling. Clinging/attachment is a stronger and more sustained type of attachment, which is said to be of four types: (1) clinging to sensuality (*rāga* in Sanskrit), which is a strong attachment to pleasing sensory objects; (2) clinging to false views and speculative theories (*dṛṣṭi* in Sanskrit); (3) clinging to faulty disciplinary codes and superstitious modes of conduct (ś*ilavrataparāmarśa* in Sanskrit); and (4) clinging to mistaken beliefs in a permanent self (ā*tmavāda* in Sanskrit), i.e., the attachment to the transitory mind and body as a real I and mine. In the context of dependent origination (PRATITYASAMUTP$\bar{\text{A}}$DA), craving (tṛṣṇā) leads to the clinging (upādāna) that nourishes the actions that will serve as the cause of “becoming/existence” (bhava), i.e., the next lifetime (Buswell and Lopez, 2013).

Here we interpret these four attachments in the context of rPD. With regard to the first attachment, if an rPD has an end, then players anticipating the end may defect in the last trial to gain even more payoff, thinking that there would be no future trial for the opponent to retaliate. Likewise, the failure of thinking beyond the end of this lifetime will not value the basis of life, let alone a commitment to help one's enemy. Thus, thinking relationships beyond this lifetime is a necessary condition to uphold the TTF-like strategies in rPD for PIC practitioners; otherwise, he or she is not really on this path.

With regard to the second attachment, accepting that one is playing rPD with a number of others indefinitely, if a practitioner sees an rPD in an invalid frame of zero-sum tug-of-war, the practitioner will misattribute the optimal strategy to those who would not work in non-zero-sum dyadic dances, resulting in cyclic conflicts i.e., *saṃsāra*. Thus, the renunciation of *saṃsāra* requires the refutation of the tug-of-war mindset and conquering-oriented strategies in the endless continuation of the dyadic relationships.

With regard to the third attachment, if one ignores the aims of the other player in rPD, he or she will over-compress a multidimensional dyadic dance into a one-dimensional tug-of-war. If a PIC practitioner is not committed to self and others' aims equally (as in conventional *bodhicitta*), he or she will not try to reframe self and others' mindset to seek the possibility of win–win scenarios in their rPD-like relationships.

With regard to the fourth attachment, as described above based on the Princeton Dictionary of Buddhism (Buswell and Lopez, 2013), any clinging will entrap someone in cyclic conflicts, which are full of wrong conduct codes (e.g., conquering-oriented strategies) and wrong views (e.g., seeing rPD as tug-of-war, based on the misbeliefs of “*us the good vs. them the evil*” and an “I” that is permanent and impervious to the consequences of one's past actions in rPD).

### 4.1. Arya Asanga's classic summary of Mahayana Buddhism

To relate dyadic active inference to Mahayana Buddhism, we introduce a relatively more elaborated summary of Mahayana Buddhism, excerpted from Arya Asanga's classic text *Bodhisattvabhumi* (Asanga, 2016), as follows (with the bracketed numbering added, to be referred to later):

“*A bodhisattva who abides in the Great Vehicle's spiritual lineage generates the thought to achieve [1]* *unsurpassed true and complete enlightenment**. The bodhisattva who has generated that thought applies him- or herself to [2]* *the attainment of one*'*s own aim and that of others**. The person who is applying him- or herself to the attainment of one's own aim and that of others finds the means by which to [3]* *avoid becoming afflicted**. The person who remains unafflicted finds the means by which to [4]* *remain free of weariness**. The person who is unwearied finds the means by which to [5]* *increase his or her roots of virtue**. The person who increases his or her roots of virtue ultimately achieves unsurpassed true and complete enlightenment. The person who is pursuing the practices that will accomplish one's own aim and that of others, pursuing the means by which one can avoid becoming afflicted, the means by which one will remain free of weariness, the means by which one will increase one's roots of virtue, and ultimately pursuing the attainment of enlightenment, at the very outset fixes his or her devotion upon the profound and extensive subjects. The person who has fixed his or her devotion upon those subjects will seek them. Having sought them, one will both teach them to others and strive to achieve them by[sic] one's own practice*.

*As one strives to achieve them, one practices in whatever way, in relation to whatever object, and for the sake of whatever purpose one ought to practice. While proceeding in that way, in relation to that object, and for that purpose, one practices in whatever way will bring about [6]* *the accumulation of merit and the accumulation of wisdom**. The person who has accumulated merit and wisdom practices the means by which to avoid abandoning samsara. While practicing in that way, one undertakes to [7]* *avoid developing the[sic] mental afflictions while remaining in* *samsara**. While practicing in that way, one undertakes to avoid becoming attached to one's own happiness. While practicing in that way, one undertakes to avoid being made weary by the suffering of samsara. While avoiding being made weary by that suffering, a bodhisattva relies upon the inner and outer bodies of teachings and becomes one who is proficient in all the bodies of teachings*.

*Having become a person who knows the various bodies of teachings, [8] one* *learns what should be taught to whom and how to go about doing* *so, thereby becoming* *one* *who knows the world**. The person who knows the bodies of teachings and who knows the world in this way seeks the Dharma in a proper manner. The person who is seeking the Dharma in this way develops the ability to remove all doubts possessed by all sentient beings. As the person who possesses this ability increases his or her merit through[sic] removing the doubts of others, he or she will complete the accumulation of merit. By increasing one's knowledge, one will also complete the accumulation of wisdom. While completing the two accumulations, one will apply oneself in a genuine manner to the practice of meditating upon the spiritual qualities that are conducive to enlightenment*.

*One will also know [9]* *the proper method of engaging in meditation**. The person who has applied him- or herself in this manner will dedicate his or her meditation practice to the attainment of the Great Vehicle's form of complete nirvana, not to the attainment of the form of complete nirvana that is pursued in the vehicles of the listeners or the solitary realizers. The person who possesses this kind of skillful means will retain in his or her mind those teachings that were uttered by all the buddhas and bodhisattvas and that were previously heard. Through the power of meditation, every aspect of the Dharma teachings that one has not previously heard will also become clear. The person who possesses this power of retention and this clarity of understanding will practice the three doors to liberation with the aim of abandoning all the obscurations. The person who practices in this way will become one who is established in[sic] the aim of [10]* *abandoning one*'*s own forms of erring belief and exaggerated* *pride as* *well* *as those of others**. This constitutes a bodhisattva's form of practice that is excellent in every respect.”* (Asanga, 2016, p. 665–667)

### 4.2. Interpreting the virtuous practices of Mahayana Buddhism in the contexts of rPD and dyadic active inference

Now we try to interpret Asanga's excellent summary of the path to enlightenment, focusing on the phrases that are underlined and numbered in the texts quoted above, to support the following hypothesis:

**Hypothesis 4**: *The virtuous practices in Mahayana Buddhism will cultivate a practitioner's commitment and capacity to steadfastly employ TTF-like strategies in rPD-like relationships*.

*1) “unsurpassed[sic] true and complete enlightenment”*

Arya Asanga used this term to refer to the direct realization of the reality of any observed object without obscurations (Asanga, 2016), which can be referred to as intuitive compassion or *ultimate bodhicitta*, as described later. The mind that allows the direct realization of reality is primordially unborn, formless, and knowing (Tenzin Gyatso the 14th Dalai Lama, 2020). Since the nature of the mind is no different between the unenlightened and the enlightened, the path to attain unsurpassed true and complete enlightenment is not a path to manufacture something that is not already present in the nature of the mind. Rather, it is a path to remove all kinds of obscurations, created by *vikalpas* and *prapañca*, which prevent the mind from seeing reality.

*2) “attainment[sic] of one's own aim and that of others”*

There are two modes of “knowing” (*jñāna in Sanskrit or yeshe in Tibetan*) that are relevant to how we interpret the attainment of one's own aim and that of others in Arya Asanga's work. The first mode of “knowing” is *intuition*, which is direct knowing unmediated by any form of light, sound, molecule, or other media. The primordial nature of the mind, which is clear and knowing, makes intuition possible. Similar to non-local information that is shared immediately and directly between the interacting events in quantum entanglement, intuitive knowledge (*vidyā in Sanskrit or rigpa in Tibetan*) is directly shared between one's own mind and that of others—for examples of a quantum approach to the brain and mind, refer to Atmanspacher (2017). Nevertheless, although intuition already affords the information necessary for someone who strives to attain his or her own aim and that of others, it is mostly obscured by mental afflictions. When a *bodhisattva* practices attaining one's own aim and that of others, he or she will need to perfect his or her intuition of self and other's aims in the process by removing all obscuring afflictions. This is one way to interpret Arya Asanga's notion that “*the bodhisattva who has generated that thought applies him- or herself to the attainment of one's own aim and that of others*.”

The second mode of “knowing” is mediated by physical forms that are imputed through *dependent designation* (*prajñaptisat*). According to the Madhyamaka school of Buddhist philosophy, the existence of a phenomenon is conceptually dependent on the designation, imputation, or convention relevant to the phenomenon under consideration. Madhyamaka Philosophy defines a person as a mere concept based on five aggregates, namely, aggregates of forms, feelings, discriminations, actions, and consciousness. The active inference framework is one of many possible approaches to model and make sense of the mediated, imputed knowledge possessed by a living organism. We speculate that there may be a functional correspondence between the notions of a person as five aggregates and that of a person as an active inference engine. In our dyadic active inference framework, when two persons (as active inference engines) are strongly coupled in dyadic interactions, the driving force of each active inference engine is to minimize its variational free energy, which can only be minimized collectively in the strongly coupled state (Ho et al., 2022). The distinction between self and other is effectively diminished in the strongly coupled state as well. As presented previously, organized life forms emerge in symbiotic ecology in which all entities are symbionts in a “community” (Ho et al., 2021, 2022). In this perspective, the application of oneself to the attainment of one's own aim and that of others is already occurring naturally during the strongly coupled dyadic interaction between any symbionts in symbiotic ecology. Besides, the strong coupling between symbionts enables the plays of cooperation and defection in a myriad of rPD-like situations to be occurring naturally in symbiotic ecology.

*3) “avoid[sic] becoming afflicted”*

For an active inference engine, to avoid becoming afflicted during dyadic interactions is to avoid being obscured by invalid beliefs in a strongly coupled state. According to Arya Asanga, erring beliefs and exaggerated pride are generated by mental fabrication or proliferation (*prapañca*) of conceptual thoughts (*vikalpas*). We have discussed the types of conceptual thoughts and levels of mental fabrication or proliferation above. Before one can actually help others attain their aims, one should first liberate him- or herself from afflictions by putting a stop to the mental fabrication of conceptual thoughts. Refer to Ch. 18 V. 5 (Nagarjuna, 1995) as quoted below.

Therefore, if one wishes to avoid afflictions, he or she ought to put a stop to actions driven by the fabrication of conceptual thoughts supported by an active inference engine, and the cessation of afflictions is accomplished by (1) preventing these conceptual thoughts from obscuring the realization of the ultimate nature of reality and (2) properly understanding that effects are an interactive product of causes by conditions.

In facing someone on the other side of a conflict, if one can stop his or her own fabrication of conceptual thoughts that conceive the self vs. other antagonisms in the death spiral of a zero-sum conflict, then one has a chance to transform conflicts into enduring peace and prosperity by choosing to employ altruistic TTF-like strategies to meet conflicts in rPD-like interactions.

*4) “remain[sic] free of weariness”*

When one wants to avoid becoming afflicted during dyadic interactions, even if he or she is strongly coupled with someone from the other side of a conflict, this person's active inference engine can maintain the strong coupling and minimize the stress that is proportional to the variational free energy generated in the dyadic interactions. This will definitely protect the person from chronic stress and weariness without disconnecting from others during dyadic interactions. Besides, when more and more conflicts are transformed into a series of interactions following altruistic TTF-like strategies, the rewards resulting from win–win scenarios will also diminish the sense of weariness.

*5) “increase[sic] his or her roots of virtue”*

By minimizing stress and weariness, one can strive to attain an understanding of others, including someone from the other side, and thus come to understand that one can increase the chance to find a win–win scenario as part of post-conflict reparation. By strengthening the skill and capacity to repair the relationship with someone from the other side of a conflict, one increases his or her roots of virtue. Here virtue refers to the potential to realize an outcome desirable for one and others alike.

*6) “the[sic] accumulation of merit and the accumulation of wisdom”*

The accumulation of merit can be interpreted as the accumulation of capacity and potential to attain win–win scenarios in social interactions. The accumulation of wisdom can be interpreted as a successive eradication of (a) the conceptual thoughts and (b) mental fabrication/proliferation that obscure the ultimate nature of reality.

*7) “avoid[sic] developing the[sic] mental afflictions while remaining in samsara”*

We interpreted this phrase as meaning that one can prevent invalid beliefs (*vikalpas*) from hijacking one's active inference engine while remaining strongly coupled with other active inference engines that are laden with invalid beliefs. Here, samsara (*saṃsāra* in Sanskrit) refers to cyclic rebirth, which is interpreted as the continuity of invalid beliefs and mental fabrication/proliferation in cyclic conflicts.

*8) “one[sic] learns what should be taught to whom and how to go about doing so, thereby becoming one who knows the world”*

At this stage, the PIC practitioner on the path to enlightenment learns how to transform conflicts into altruistic peace in numerous kinds of rPD-like situations in the “real” world. She or he will become one who knows the world because she or he cannot accomplish the transformation of the conflicts without knowing what his or her counterpart's intents and plays are.

*9) “proper[sic] method of engaging in meditation”*

According to Arya Asanga:

“*It is a virtuous one-pointedness of mind that is possessed by bodhisattvas and is preceded by listening to and reflecting upon the collection of bodhisattva scriptures. It can be either ‘**mundane*'* or ‘**transcendent*'* in nature. [Moreover] it is a state of mental stability that pertains to quiescence or insight, or both of them in that it constitutes a path in which the two [forms of meditation] are practiced in combination. This should be understood as the essence of the ‘meditative absorption' that is practiced by bodhisattvas*.” (Asanga, 2016, p. 343)

Here, “meditative absorption” refers to a state in which the practitioner meditates correctly on an object and steadfastly holds the recollection of the object single-pointedly; “mundane” meditation refers to a form of insight meditation that refines the level of consciousness from being coarser to being more tranquil and it results in “freedom from attachment” to any form of illusory egoistic existence (as if one only existed in a weakly coupled state independent of others and the world); “transcendental” meditation refers to a non-conceptual state of mind that is free of both conceptual thoughts (*vikalpas*) and mental fabrication/proliferation (*prapañca*); thus, such transcendental meditation is an antidote for all forms of the illusory egoistic notion of existence (Asanga, 2016, p. 343).

A seminal example of proper meditation for a bodhisattva's practice is known as *lojong* (or *blo sbyong* in Tibetan). According to the Princeton Dictionary of Buddhism, *lojong* is a form of intuitive compassion meditation that specifically trains (*sbyong*) a practitioner to comprehend (*blo*) the ultimate nature of the mind and all phenomena. *Lojong* emphasizes how to see conflicts (and other circumstances that are ordinarily upsetting or depressing) as occasions for happiness in the perspective of dependent origination, e.g., thinking that adversities or difficulties are exhausting negative karmic results of one's own non-virtuous actions in the past. Specific practices include how to transform self-cherishing attitudes into cherishing others, by contemplating the illusory nature of the self, the faults in self-cherishing, and the benefits that flow from cherishing others (Buswell and Lopez, 2013).

*Lojong* training is based primarily on the techniques for (1) equalizing the attunement to self and others and (2) exchanging self and others by taking other's suffering and giving them self's happiness (Buswell and Lopez, 2013). The first meditative technique refers to three levels of equality that a *lojong* practitioner has to cultivate first as prerequisites (Rimpoche, 2007), as follows:

1. First, dwelling on equality in wishing all beings to be happy and free from suffering
2. Second, dwelling on equality in developing equanimity to friends and enemies in one's own responses, i.e., inhibiting one's attraction to friends and repulsion from enemies
3. Third, dwelling on equality in *identifying with* friends and enemies *equally* as if their sufferings are one's own

The second meditative technique refers to the give-and-take (*tonglen, or gtong len* in Tibetan) to take others' suffering and give one's own happiness. It aims to transform how one relates to the suffering that he or she experiences, from seeing adversities as unwanted stressors to welcoming them as treasury, because these sufferings can (a) exhaust negative consequences of past non-virtuous deeds, (b) enhance one's renunciation of such non-virtues, (c) open one's mind to change, (d) cultivate compassion for others who shared the same experiences, and (e) strengthen the aspiration for realizing the ultimate nature of mind and all phenomena for the benefits of all—as stated in Arya Shantideva's Guide to a Bodhisattva's Way of Life (*Bodhisattvacharyavatara* in Sanskrit), Ch. 6, V.21:

*Furthermore, suffering has good qualities: through being disheartened with it (cyclic existence is renounced), arrogance is dispelled, compassion arises for those in cyclic existence, evil (non-virtue) is shunned, and joy is found in virtue*. (Shantideva, c. 700/1979)

The single-pointed “mundane” and “transcendental” meditations can be described according to Geshe Chekawa's Mind Training in Seven Points (Tulku, 1998), as follows (original texts in *italics*, with some interpretive notes added in parentheses):

*Training in relative bodhicitta*

*Put all the blame on the one* (eradicate ego-grasping *vikalpas*).
*Meditate on everyone as kind* (as in symbiotic relationships).
*Train alternately in the two, taking and giving* (*tonglen*).
*Begin taking with yourself* (taking one's own suffering as a valuable steppingstone on the path).
*Mount the two upon the breath* (this would need to be taught by a qualified teacher).
*There are three objects* (friend, enemy, and stranger), three *poisons* (greed, hatred, and wrong view), *and three roots of virtue* (virtuous practices that can transform the poisons).
*The following are brief instructions for the post-meditation period* (during everyday activities):
*Be mindful in order to admonish yourself* (the tug-of-war mindset in perceiving conflicts).
*Train yourself with the verses during all activities* (especially when arriving at a conflict).

*Training in ultimate bodhicitta*

*Having attained stability, be shown the secret* (of the ultimate nature of reality).
*Consider phenomena to be like a dream* (dependent origination).
*Analyze the nature of unborn awareness* (the nature of mind as clear and knowing).
*Even the antidote itself is naturally free* (of *vikalpas*).
*Focus on the nature of the basis of all, the entity of the path* (the basis of all refers to emptiness, i.e., Effect = Cause × Condition, as we postulated here)
*Between sessions, be an illusionist* (all phenomena are effects of interactive products of cause-by-condition interactions).

We further discuss the symmetry between *lojong* meditation and rPD later.

*10) “abandoning[sic] all the obscurations... abandoning one's own forms of erring belief and exaggerated pride as well as those of others”*

Arya Asanga suggested that erring beliefs and exaggerated pride effectively obscure the ultimate nature of reality. Thus, to achieve unsurpassed enlightenment, one has to completely abandon any forms of erring belief and exaggerated pride. The erring beliefs are interpreted here as those invalid beliefs that hijack one's active inference engine. The exaggerated pride is interpreted here as the self-centered beliefs about oneself that are impervious to the updating of the prior beliefs despite being strongly coupled with another person's active inference engine, resulting in zero-sum conflicts with others and the attitude of *conquering* others, rather than transforming conflicts into enduring peace and prosperity.

### 4.3. Symmetry across the domains of rPD, dyadic active inference, and Mahayana Buddhism

We postulate that intuitive compassion is an inherent function—therefore a functional synonym—of *ultimate bodhicitta*, for the following two reasons. First, in Madhyamaka Philosophy, *ultimate bodhicitta* refers to the direct realization of emptiness (ś*unyatā* in Sanskrit) as the ultimate truth (Buswell and Lopez, 2013). A direct realization is, by definition, intuitive, i.e., direct perception unmediated by any form of media. According to Arya Nagarjuna (circa 150–250 CE), the emptiness that *ultimate bodhicitta* directly realizes is the dependent origination in all phenomena:

“Neither from itself,

Nor from another,

Nor from both,

Nor without a cause,

Does anything whatever, anywhere arise.” (Nagarjuna, 1995) Ch. 1 V. 1

Previously, we explained how Arya Nagarjuna's reasoning on emptiness is equivalent to a formal expression of the notion that “effect is an interactive product of cause by condition” (Ho et al., 2022):


$$\begin{array}{l} \text{Effect}=\text{Cause}\times \text{Condition} \end{array}$$

Notably, this expression is also compatible with the *interactive* payoff matrix in rPD, supporting the conjecture that realizing the ultimate nature of reality will help a player to play optimally in rPD.

Second, the direct realization of the ultimate truth is compassionate in nature because it automatically eradicates *vikalpas* and *prapañcas* that cause all afflictions, according to Arya Nagarjuna:

“Action and misery having ceased, there is nirvana.

Action and misery come from conceptual thought (*vikalpas*).

This comes from mental fabrication (*prapañca*).

Fabrication ceases through emptiness (ś*unyatā*).” (Nagarjuna, 1995) Ch. 18, V. 5

To highlight intuitive compassion as a functional synonym of ultimate *bodhicitta*, we refer to the preamble and Verses 59–70, in *italics*, of Arya Nagarjuna's Commentary on the *Bodhicitta* (*Bodhicittavivāraṇa* in Sanskrit) (Nagarjuna, 2006), with our own interpretive notes added in brackets, as follows:

“*Devoid of all real entities; Utterly discarding all objects and subjects, such as aggregates, elements, and sense-fields; due to sameness of selflessness of all phenomena, one's mind is primordially unborn; it is in the nature of emptiness. Just as the blessed Buddhas and the great bodhisattvas have generated the mind of great awakening, I too shall, from now until I arrive at the heart of awakening, generate the awakening mind in order that I may save those who are not saved, free those who are not free, relieve those who are not relieved, and help thoroughly transcend sorrow [in] those who have not thoroughly transcended sorrow. Those bodhisattvas who practice by means of the secret mantra, after having generated awakening[sic] mind in terms of its conventional aspect in the form of an[sic] aspiration, must [then] produce the ultimate awakening mind through the force of meditative practice*.” (Nagarjuna, 2006)

Verse 59.

*Starting with ignorance and ending with aging*

*All processes that arise from*

*The twelve links of dependent origination*

*We accept them to be like a dream and an illusion*.

[The ignorance here refers to the obscuration of the ultimate nature of reality; the twelve links of dependent origination, therefore, can be understood as the working of *vikalpas* and *prapañcas* that hijack an active inference engine to cause malfunctioning in dyadic interactions.]

Verse 60.

*This wheel with twelve links*

*Rolls along the road of cyclic existence*

*Outside this, there cannot be sentient beings*

*Experiencing the fruits of their deeds*.

[A person whose *vikalpas* and *prapañcas* obscure the nature of rPD-like dyadic interactions will be trapped in cyclic conflicts due to the misbelief of “*us the good vs. them the evil*.”]

Verse 61.

*Just as in dependence upon a mirror*

*A full image of one's face appears*

*The face did not move onto the mirror*

*Yet without it, there is no image [of the face]*.

[Here, the relationships among (a) a person, (b) one's face, (c) the mirror, and (d) the image of the face serve as a metaphor for the relationships among (a′) an observer (subject), (b′) an object to be observed, (c′) an interaction between the observer and the object being observed, and (d′) what the observer perceives the observed object to be *like* (*qualia*), respectively. This verse is a metaphoric expression of the notion that Effect = Cause × Condition, wherein Effect refers to “the image of a face,” i.e., the qualia that the observer perceives the face to be like (“face-ness”), Cause refers to the person in “one's face,” i.e., the observer, Condition refers to the “face” to be observed, and the operation “X” refers to “the mirror,” i.e., the interaction. This metaphoric expression describes the nature of the mind, as further discussed later. The metaphor also implicates the meaning of intuition in intuitive compassion, as the observer's perception of the observed (“the image of face”) is directly caused by the interaction (“mirror”) between the observer and the object to be observed (“the face”), unmediated by any third-party media.]

Verse 62.

*Likewise, aggregates recompose in a new existence*

*Yet the wise always understand*

*That no one is born in another existence*

*Nor does someone transfer to such existence*.

[In the context of conventional phenomena, such as dyadic interactions between two active inference engines in rPD, this verse may be understood in the following way: the re-composition of aggregates in a new existence refers to a new state of a player, i.e., an active inference engine after a dyadic interaction is neither causeless nor the same as the original state before the interaction. Thus, in the context of rPD, this particular verse is consistent with the expression of Payoff_(Alice)_ = Play_(Alice)_ × Play_(Bob)_, wherein the new state of Alice is an interactive product, not a linear transformation, of Alice's or Bob's original state.]

Verse 63.

*In brief, from empty phenomena*

*Empty phenomena arise*

*Agent, karma, fruits, and their enjoyer –*

*The conqueror taught these to be [only] conventional*.

[One may interpret this verse as suggesting that from one dyadic interaction, the next arises, with all parts of the interactions following the expression of emptiness, i.e., Effect = Cause × Condition. In the context of rPD, agent, karma, fruits, and their enjoyer in this particular verse refer to the active inference engine of a player Alice (agent), her action Play_(Alice)_ (karma), her Payoff_(Alice)_ (fruits), and the operation “X” (enjoyer) in the expression of Payoff_(Alice)_ = Play_(Alice)_ × Play_(Bob)_, respectively. Each element in this expression exists conventionally according to its name and form from the perspective of dependent designation, without having any non-relational, intrinsically independent essence.]

Verse 64.

*Just as the sound of a drum as well as a shoot*

*Are produced from a collection [of factors]*

*We accept the external world of dependent origination*

*To be like a dream and an illusion*.

Verse 65.

*That[sic] phenomena are born from causes*

*Can never be inconsistent [with facts]*

*Since the cause is empty of cause*

*We understand it to be empty of origination*.

[These two verses point out that in the expression of Effect = Cause × Condition, any Cause and Condition themselves are the interactive products of the previous Cause and Condition, and therefore, none of them are born without interactions between their own cause and condition, and “empty of origination” refers to the fact that they do not exist intrinsically.]

Verse 66.

*The non-origination of all phenomena*

*Is clearly taught to be emptiness*

*In brief, the five aggregates are denoted*

*By [the expression] “all phenomena.”*

[Five aggregates of a person [forms, feelings, discriminations, actions, and consciousness] may refer to the active inference processes that constitute person–environment interactions. When an active inference engine (the observer) interacts with an object in a strongly coupled state, both the observer and the observed (the object) become the interactive products of causes by conditions involved in their interaction. The same principle is applied to those interactions among the sensory, active, and internal states within the active inference engine. In this sense, all information processed in person–environment interactions is *relational* in nature. Using the metaphor described above in Verse 61, the appearance (qualia) of the observed object (i.e., “the image of the face in the mirror”) is the information extracted from the object (i.e., “one's face in front of the mirror”) by the observer (i.e., “the person seeing the image”) after the interaction (e.g., “the mirror” between the observer and the observed object) occurs. For detailed discussion based on Physics, Biology, and Psychology on this notion, refer to our previous work (Ho et al., 2022).]

Verse 67.

*When the [ultimate] truth is explained as it is*

*The conventional is not obstructed*

*Independent of the conventional*

*No [ultimate] truth can be found*.

[One way to interpret this verse is that due to the unity of conventional truth and ultimate truth based on Effect = Cause × Condition, the wisdom realizing the ultimate truth and compassion traversing the conventional truth are inseparably united in the *ultimate bodhicitta*—intuitive compassion—that directly realizes such unity, as described in the following three verses 68–70.]

Verse 68.

*The conventional is taught to be emptiness*

*The emptiness itself is the conventional*

*One does not occur without the other*

*Just as [being] produced and impermanent*.

Verse 69.

*The conventional arises from afflictions and karma*

*And karma arises from the mind*

*The mind is accumulated by the propensities*

*When free from propensities it's happiness*.

Verse 70.

*A happy mind is tranquil indeed*

*A tranquil mind is not confused*

*To have no confusion is to understand the truth*

*By understanding the truth, one attains freedom*.

### 4.4. Summarizing the symmetry in Eight Verses

The symmetry across the three domains of Mahayana Buddhism, dyadic active inference, and rPD can be summarized in terms of Eight Verses of *lojong* Mind Training (*lojong* *tsikgyema* in Tibetan) composed by Geshe Langri Thangpa (1054–1123), as follows (with our own interpretive notes added in brackets following each verse):

1. *By thinking of all sentient beings*
*As more precious than a wish-fulfilling jewel*
  *For accomplishing the highest aim*,
  *I will always hold them dear*.
  [Generating the conventional bodhicitta to fulfill the aims of self and others equally.]
2. *Whenever I'm in the company of others*,
  *I will regard myself as the lowest among all*,
*And from the depths of my heart*
  *Cherish others as supreme*.
  [Whenever in dyadic relationships, give up any self-centered unidimensional view to make it possible to frame all dyadic interactions in an rPD-compatible multidimensional frame. This is the basis of the first level of equality.]
3. *In my every action, I will watch my mind*,
  *And the moment destructive emotions arise*,
  *I will confront them strongly and avert them*,
  *Since they will hurt both me and others*.
  [Refrain from being influenced by any conceptual thoughts that misperceive conflicts in terms of the tug-of-war metaphor. Prepare to identify with others without being carried away by self or others' conceptual thoughts and negative emotions. This is the basis of the second level of equality.]
4. *Whenever I see ill-natured beings*,
  *Or those overwhelmed by heavy misdeeds or suffering*,
  *I will cherish them as something rare*,
  *As though I'd found a priceless treasure*.
  [Prepare to perform TTF-like strategies when facing conflicts with a stranger with equality in fulfilling the aims of self and others. This is the first one-third of the basis of the third level of equality.]
5. *Whenever someone out of envy*
  *Does me wrong by attacking or belittling me*,
  *I will take defeat upon myself*,
  *And give the victory to others*.
  [Prepare to perform TTF-like strategies when facing conflicts with an enemy with equality in fulfilling the aims of self and others. This is the second one-third of the basis of the third level of equality.]
6. *Even when someone I have helped*,
*Or in whom I have placed great hopes*
  *Mistreats me very unjustly*,
  *I will view that person as a true spiritual teacher*.
  [Prepare to perform TTF-like strategies when facing conflicts with a friend with equality in fulfilling the aims of self and others. This is the last one-third of the basis of the third level of equality.]
7. *In brief, directly or indirectly*,
  *I will offer help and happiness to all my mothers*,
*And secretly take upon myself*
  *All their hurt and suffering*.
  [Without announcing in any way what would undermine the deployment of TTF-like strategies in rPD, practice a single-pointed meditation to identify with others' sufferings arising in the conflicts and use the conflicts to eradicate conceptual thoughts and suspend mental fabrication with a steadfast commitment to benefitting self and others equally.]
8. *I will learn to keep all these practices*
  *Untainted by thoughts of the eight worldly concerns*.
  *May I recognize all things as like illusions*,
  *And, without attachment, gain freedom from bondage*.
  [Keep the practice of conventional bodhicitta free of the tug-of-war metaphor in which zero-sum conflicts are bound to happen on a single dimension with positive and negative payoffs measured in the forms of experiencing pleasant/unpleasant feelings, getting gain/loss of resources, being liked/disliked in relationships, and having good/bad reputations, due to the misperception of the payoff matrix based on the difference between the players' plays, e.g., Payoff_Self_ = Play_Self_ – Play_Other_. Instead, focusing on the ultimate nature of the dyadic relationships in rPD-like dances, one can rest with the understanding of the payoff being the effect of the interactive product of cause by condition, e.g., Payoff_Self_ = Play_Self_ × Play_Other_.]

### 4.5. A summary of the path of intuitive compassion

We summarize the opposite directions on PIC as two incompatible paths in Table 4. The gist of these opposing directions can be captured in the two sets of verses as follows, either

Love first.
Inquire later.
Inquire to love, Not to conquer.

Or

Exploit first.
Manipulate later.
Manipulate to exploit, Not to love one another.

**Table 4:** Summary for two incompatible directions that bifurcate at each conflict as a stepping stone on the path of a dyadic relationship.

**Basis of the path**	**Path of intuitive compassion**	**Path of cyclic conflicts**
	**Valid views of the nature of reality**	**Wrong views of the nature of reality**
View on life	Four noble truths: 1. Life with cyclic conflicts is suffering 2. Causes of suffering 3. Cessation of suffering is nirvana (enduring peace) 4. Path to nirvana	Four non-virtuous misbeliefs 1. Life is winner-takes-it-all 2. Cause of winner-takes-it-all 3. Never being caught is the best 4. Path to never being caught
Weighing of one's own and opponent's aims	Equality in wishing to fulfill self and other's aims	One's own aim overweighs the opponent's
State of social relationship	Strongly coupled state (see Figure 3)	Weakly coupled state (see Figure 4)
View of one's own and opponent's payoff in social interactions	Payoff_(self)_ = Play_(self)_ × Play_(opponent)_ Payoff_(opponent)_ =Play_(opponent)_ × Play_(*self*)_	Payoff_(self)_ = Play_(self)_ – Play_(opponent)_ Payoff_(opponent)_ =Play_(opponent)_ – Play_(*self*)_
Perception of social relationships	Endless rPD-like multidimensional dances	Opportunity to exploit others and run away in one-dimensional tug-of-wars
Perception of the purpose of one's own actions	To reciprocate what the opponent did or to repair damages in social relationships	To conquer or exploit social relationships for one's selfish gains
Perception of future	Future is a relational consequence of current dyadic interactions	Future is a chance to escape from obligations or consequences of one's past actions
Summary verse	Love first Inquire later Inquire to love Not to conquer	Exploit first Manipulate later Manipulate to exploit Not to love one another

There is no doubt that conflicts are unpleasant and that no one would enjoy any aversive experiences in suffering. Whenever a conflict happens in a dyadic relationship, a person guided by PIC would realize that the conflict serves as a stepping stone on a bidirectional path and he or she will decide which direction to go on the path. The foundation for treating conflicts as stepping stones to enduring peace lies in the recognition of the nature of suffering and the values thereof. When a conflict occurs, a practitioner of PIC will see suffering in the conflict as a blessing in disguise. Of course, many inquiries would need to be conducted and they will have to be answered pointedly and properly, if one wants to figure out how to transform conflicts into enduring peace by properly engaging one's counterparts in conflicts in a series of interactions, realizing that we are all playing non-zero-sum rPD, rather than zero-sum games.

For partners in rPD-like relationships, such as close relationships, practicing PIC can help repair the relationships by (1) transforming win–lose or lose–win conflicts into win–win scenarios and (2) reducing their resentment toward each other after encountering defections. For example, *lojong* meditation can strengthen the commitment to employing TTF-like strategies to strive for win–win scenarios, such that the partners will be nice (start the rPD with cooperation and never defect first) and learn how to suspend his or her conceptual thoughts so that they can recognize that the best practice in their relationship is to follow TTF-like strategies. By practicing *tonglen* (self-other exchange by taking other's suffering to oneself and giving one's own happiness to others), one will take in his or her partner's defection by reciprocating with either retaliation (to defect without generating any resentment or blame) or forgiveness (to cooperate without acting out of ignorance or greed) in the following trial.

## 5. Conclusion

Conflicts are ubiquitous, and they entail sufferings for all parties involved. Compassion, i.e., a wish to relieve others from suffering, is a commitment to face conflicts and sufferings in a mindset that makes it possible to transform them into enduring peace and prosperity. We introduced PIC to highlight the symmetry across the domains of (a) rPD, (b) dyadic active inference, and (c) Mahayana Buddhism, in their theoretical and practice aspects alike, as geometrically represented in Figure 2.

In the theoretical aspect, the symmetry lies in the recognition that the elements of interest operating in these domains are the effects of the interactive product of cause by condition: in rPD, the elements—the players' payoffs—are the effects of an interactive product of their actions; in dyadic active inference, the elements—intersubjectivity and stress-reduction—are the effect of an interactive product of two strongly coupled active inference engines; in Mahayana Buddhism, the elements—all phenomena including the actor, the receiver, and the object/energy being exchanged between them—are the effects of an interactive product of causes and conditions.

In the practice aspects, the symmetry lies in those strategic techniques that are commonly shared in the proven strategies that can yield optimal outcomes in each of these domains: in rPD, the technical features are being nice, reciprocating, forgiving, and non-envious in TTF-like strategies; in a dyadic active inference, the strategic techniques are dyadic concepts, e.g., mutuality, reciprocity, attunement, contingency, reparation, matching, mirroring, coordination, and synchrony in dyadic interactions, which can be boosted to become conflict-proof by dyadic interventions targeting problems that may allow conflicts to impair dyadic interactions (*deficient relational benevolence, under-coupling, and over-mentalizing*); in Mahayana Buddhism, the strategic techniques are often described as virtuous practices of generosity, ethics, patience, non-weariness, and meditations, with wisdom being the inseparable companion of these practices.

Conflict as a point of bifurcation, like a stepping stone, is also embedded in the symmetry across these domains. In rPD, conflicts are those win–lose or lose–win scenarios to be transformed into an enduring win–win or lose–lose scenarios depending on the strategies. In dyadic active inference, conflicts are collective surprises between two strongly coupled active inference engines that would need to be minimized. In Mahayana Buddhism, conflicts and sufferings of one's own or others' are equally valuable for the practitioners to transform, like the poisons that can make a peacock even more splendorous. Likewise, compassion in the face of conflicts is also part of the symmetry. We postulated that compassion reflects the conflict-proof commitment to choosing a non-zero-sum frame, as opposed to a zero-sum one, to fulfill self and others' aims equally in all these domains.

A caveat is that this commitment to compassion is not the same as unconditional cooperation with the opponent. The reciprocity, as one of the strategic techniques in these domains, cannot be overly diminished by always cooperating unconditionally even after the opponent's defection in the last move. Most of the time, the opponent's defection should be retaliated by defections, except for occasional forgiveness that comes at an auspicious timing (to be discerned and determined by someone with intuitive compassion). While such reciprocity means that retaliation is part of the optimal TTF-like strategies, it does not mean that the retaliation must be violent in nature. For example, Mahatma Gandhi led the successful campaign for India's independence from British rule by holding a steadfast non-violence principle in retaliating against the opponent's oppression with a non-violent movement of non-cooperation. In a way, the UK–India interactions at that time may serve as a good case for demonstrating that two players in conflicts can find an enduring win–win solutions in the real world. When all things considered, both parties saw that the “pie” of mutual cooperation, in the form of India's independence, is bigger than what it was otherwise at the time of post-World War II.

Thus, though violence is common in the time of conflict, especially when one side wants to exterminate the other side for good, this only calls for more intuitive compassion to creatively make the pie of mutual cooperation bigger than cyclic conflicts (i.e., 2R > G + U) and to persistently take the time for conflicts to be transformed into enduring peace eventually. In doing so, the retaliation part of TTF-like strategies, seemingly violent or not, should be done with a steadfast commitment to eventual mutual cooperation and enduring peace.

“*Non-violence takes a long time*,” said the 14th Dalai Lama.

“*Do we have the time, Holiness?*” his bodyguard asked.

“*I've never known*,” said the 14th Dalai Lama.

~ *Kundun, the film* (Scorsese, 1997)

Indeed, no one would know whether humanity has enough time to dissolve all the conflicts that we face today. Nevertheless, Bodhisattvas seem not concerned about time because their great compassion enables them to be willing to spend three infinite eons or longer to complete the path to enlightenment (Buswell and Lopez, 2013). Perhaps in their intuitive compassion, they can see the transient nature of conflicts and the destined peace and prosperity all along, just like playing rPD games with a proven winning strategy steadfastly. In dedicating their virtuous practices, Bodhisattvas would pray as what Arya Shantideva did in *Bodhisattvacharyavatara* (Shantideva, c. 700/1979), Ch. 10, V.55,

*For as long as space endures*

*And for as long as living beings remain*,

*Until then may I too abide*

*To dispel the misery of the world*.

## Data availability statement

The original contributions presented in the study are included in the article/supplementary material, further inquiries can be directed to the corresponding author.

## Author contributions

SH is the principal developer of the theoretical framework and hypotheses, the writer of the manuscript, and created the figures in the study. YN has collaborated with SH in developing and refining the theoretical framework presented in this article. He also co-wrote the manuscript. JS co-wrote the manuscript and supported the research involved in the manuscript. All authors contributed to the article and approved the submitted version.
